# 

**Published:** 1900-03-17

**Authors:** 


					TUB HOSPITAL. "|The standard of highest purity."? Lancet, March 17tii, 1900.
CADBURY'S U ti CADBURY'S
COCOA jlS Mk'm COCOA
ABSOLUTELY PURE. NO DRUGS OR ALKALIES USED.
T'CLasco>r Western Infi'rmah
No. 703. Vol. XXVII. Saturday,
March 17, 1900.
;S?bsoSt sg?"'] PRICE TWOPENCE.

				

